# Effects of Intermittent Versus Continuous Small-Sided Games on Athletic Performance in Male Youth Soccer Players: A Pilot Study

**DOI:** 10.3390/life15030364

**Published:** 2025-02-26

**Authors:** Zarife Pancar, Mehmet Kaan Akay, Muhammet Taha İlhan, Emre Karaday, Burak Karaca, Mustafa Sencer Ulema, Ali Muhittin Taşdoğan, Yücel Makaracı, Francisco Tomás González-Fernández

**Affiliations:** 1 Department of Physical Education and Sports, Faculty of Sports Science, Gaziantep University, Gaziantep 27350, Turkey; 2 Department of Physical Education and Sports, Gaziantep University, Gaziantep 27350, Turkey; 3 Faculty of Health Sciences, Hasan Kalyoncu University, Gaziantep 27000, Turkey; 4 Department of Coaching Education, Faculty of Sports Sciences, Karamanoglu Mehmetbey University, Karaman 70100, Turkey; 5 Department of Physical Education and Sports, Faculty of Sport Sciences, University of Granada, 18071 Granada, Spain

**Keywords:** interval training techniques, spatially constrained play, physical conditioning, young athletes

## Abstract

Adjusting the design of small-sided games and selecting the appropriate format can significantly enhance training outcomes and player development in soccer. The aim of the study was to compare the effects of intermittent small-sided games (ISSG) and continuous small-sided games (CSSG) on athletic performance metrics in male soccer players. This pilot study was conducted using a parallel group pre-test and post-test design, with 16 male youth soccer players randomly assigned to two groups: the ISSG group (*n* = 8, Mean age = 16.50 ± 0.53 years) and the CSSG group (*n* = 8, Mean age = 16.63 ± 0.52 years), ensuring a balance in pre-test performance and playing positions. The ISSG protocol began with 4 min sets in week one, progressively increasing to 7 min by week four, with 2 min rest intervals. The CSSG protocol involved continuous play, starting at 16 min and progressing to 28 min over the same period. Players underwent pre- and post-tests, with a 4-week training period. Performance metrics assessed included body composition, a 30 m sprint test, agility, horizontal jump, aerobic and anaerobic capacity, and static balance. Training intensity was monitored using the CR-10 Borg Rating of Perceived Exertion scale throughout the training period. Both groups exhibited improvements in horizontal jump, agility, aerobic and anaerobic power, and static balance, with no significant between-group differences. Sprint performance did not significantly improve in either group. The CSSG group reported higher RPE values and experienced a reduction in body mass index. Our findings demonstrate that both ISSG and CSSG resulted in similar improvements in athletic performance metrics in male youth soccer players. Coaches and practitioners can choose the most appropriate training method based on specific objectives, session duration, and player fatigue levels, thereby optimizing training outcomes.

## 1. Introduction

In order to achieve success in soccer, players must possess a high level of physical, technical, and tactical abilities [1,2,3,4]. The dynamic nature of the sport demands athletes to be not only physically fit but also skilled in making quick decisions and executing complex maneuvers under pressure [5,6]. To address these multifaceted demands, small-sided games (SSGs) have emerged as a contemporary training approach that enhances these attributes more effectively than traditional training methods [7,8]. SSGs involve modified games with adjusted rules, played in compact areas with fewer participants than official matches [9,10,11]. This format allows for increased ball touches, more frequent interactions, and a greater emphasis on tactical awareness, making it an ideal training tool for football players [12]. The effectiveness of SSGs in refining technical and tactical skills under match-specific conditions has been widely recognized in the literature [7,8,9]. By simulating the pressures and dynamics of real match situations, SSGs offer players the opportunity to develop decision-making skills, improve spatial awareness, and enhance teamwork [13]. Nevertheless, an ongoing debate exists regarding the optimal use of SSGs to enhance the physical, technical, and tactical proficiencies of soccer players [12]. This debate is driven by the variability in training outcomes, which depend on the specific design and implementation of SSGs.

Numerous variables can impact the training intensity during SSGs, including the number of actions permitted, player quantity, and pitch dimensions, which can significantly influence physical and technical/tactical performance outcomes [14,15]. For instance, smaller pitch sizes may lead to increased ball possession and more frequent interactions, thereby enhancing players’ technical skills. Conversely, larger pitches may require players to cover more ground, thereby improving their aerobic capacity [15]. Additionally, the distinction between intermittent small-sided game (ISSG) and continuous small-sided game (CSSG) can also influence these characteristics [14,16]. While both ISSG and CSSG are commonly employed to enhance the aerobic endurance performance of soccer players [17,18], studies have explored the effects of these formats on various parameters. Although previous studies [16,19] suggest that CSSG may impose greater cardiovascular demands due to the absence of rest intervals, overall improvements in aerobic capacity have been reported as similar for both methods. However, the combined effects of both methods on physical fitness characteristics, without emphasizing significant differences in aerobic capacity, still require further clarification. Daryanoosh et al. [16] investigated the impacts of 8-week ISSG and CSSG interventions on young soccer players during the preseason. The study found no significant difference in aerobic power between the groups, with the ISSG group demonstrating greater improvements in anaerobic power. Additionally, the CSSG group exhibited higher mean heart rates and ratings of perceived exertion (RPE), while the ISSG group recorded higher maximum heart rates. These results suggest that the effects of ISSG and CSSG on physical fitness characteristics may vary, warranting further research to elucidate these differences and assist coaches and practitioners in tailoring SSG programs to individual needs and objectives [20,21].

The optimization of SSGs stands as a pivotal strategy for enhancing players’ physical, technical, and tactical proficiencies [22]. The effectiveness of SSGs in replicating match-specific conditions and fostering skill development has been widely acknowledged in the literature [17,18,23]. However, the effective implementation of SSGs depends on a nuanced understanding of the variables that influence training intensity and performance outcomes within these game formats [24,25]. This intricate interplay underscores the need for coaches and practitioners to carefully manipulate these variables to optimize training stimuli and desired outcomes. Notably, studies have highlighted differences in fatigue indices, heart rate profiles, and perceived exertion levels between ISSG and CSSG sessions, underscoring the need for tailored approaches based on specific training objectives and player characteristics [26,27,28,29]. For instance, players focusing on anaerobic capacity may benefit more from ISSG, while those targeting aerobic endurance might find CSSG more advantageous. Such tailored approaches not only enhance the effectiveness of training but also help prevent overtraining and reduce the risk of injury [30,31].

In summary, strategically manipulating variables in SSG design, alongside a thoughtful selection of intermittent or continuous formats, offers significant potential to optimize training outcomes and player development in soccer. By exploring the intricate interplay of these factors and their effects on performance, coaches can refine their methodologies to elevate players toward peak athletic excellence. Considering the limited sample size, this research was designed as a pilot study to provide preliminary insights into the effects of different SSG formats on athletic performance in youth soccer players. The primary objective of this study, therefore, was to compare the impact of ISSG and CSSG on physical fitness characteristics, including aerobic power, agility, speed, horizontal jump, static balance, and repeated sprinting ability in young male soccer players. Another objective of the study was to assess the training intensity associated with both approaches (i.e., ISSG and CSSG) using the RPE scale. We hypothesized that both ISSG and CSSG would result in similar improvements in athletic performance metrics, with RPE levels being higher in the CSSG group compared to the ISSG group, due to the absence of rest intervals, in male youth soccer players.

## 2. Materials and Methods

### 2.1. Study Design

The present pilot study was conducted from March to May 2024. Prior to the experiment, the soccer players underwent a 4-week familiarization period with the test protocol. This familiarization phase aimed to ensure that participants were fully accustomed to the testing procedures and training interventions. During this period, all participants were introduced to the details of each test, such as the 30 m sprint, Illinois agility test, Yo-Yo test, and other fitness assessments. Each test was demonstrated and practiced multiple times to ensure accurate execution and increase participants’ confidence. Additionally, low-intensity SSGs were incorporated to familiarize participants with the training protocols. The familiarization sessions were held twice a week, with each session lasting approximately one hour. The intensity was progressively increased over the weeks, beginning with low-intensity activities and gradually incorporating more challenging tasks. Dynamic warm-up routines, including low-intensity running and sport-specific stretching, were performed before each session to reduce the risk of injuries. The study lasted a total of 6 weeks, consisting of 1 week of pre-testing, 4 weeks of training intervention, and 1 week of post-testing. Body composition, the 30 m sprint test, Illinois agility test (IAT), horizontal jump test (HJ), Yo-Yo Intermittent Recovery Test-1 (YYIRT-1), repeated sprint test (RAST), and static balance test (SB) were measured before the experimental intervention began. The CR-10 Borg perceived difficulty scale was also introduced. Table 1 shows the timeline of the study.

A randomized parallel two-group, pre-post design was used, with physical fitness tests performed before (pre-intervention) and after (post-intervention) the 4-week intervention period. The soccer players were randomly assigned to two groups (ISSG and CSSG) using a computer-generated randomization method. To ensure balance in physical fitness and playing characteristics, stratification was applied prior to randomization based on Yo-Yo test results and soccer-specific positions. Both groups followed the SSG design specific to their group, with two sessions per week (at least 48 h between sessions) over 4 weeks. This stratified randomization ensured that both groups were balanced in terms of fitness levels (based on Yo-Yo test results) and playing positions while maintaining the random allocation of players to groups. As the study was conducted during the pre-season, the players did not participate in any other training programs. Players were ranked based on their Yo-Yo Intermittent Recovery Test results and evenly distributed into the ISSG and CSSG groups to ensure balanced baseline physical and performance characteristics. This stratified randomization approach minimized potential confounding factors, such as disparities in aerobic capacity and physical fitness levels, while maintaining randomization principles through unbiased allocation within defined strata. This method enhanced the internal validity of the study by reducing variability caused by differences in initial fitness levels.

After the 4-week training intervention, the same tests were repeated under similar conditions. All training sessions and tests were performed on an artificial turf soccer pitch and at the same time of day (16–18 p.m.). In both groups, SSGs were played as four players against four players (4 vs. 4) on a 25 × 32 m pitch. In the SSGs, no goalkeepers or target players were used, and the focus was on a ball possession game. According to this game, a player could touch the ball only two times at a time and if he touched the ball more than two times, the ball was passed to the opposite team and the opponent gained one point. The ball possession game was chosen because it provides an effective platform for simultaneously developing players’ technical, tactical, and physical skills. This type of game emphasizes decision-making, spatial awareness, and teamwork critical attributes for football performance. Additionally, a standardized ball possession format was used to ensure consistent conditions for both groups, allowing for an equitable comparison of the ISSG and CSSG protocols.

To ensure equivalence in technical ability between the groups, each team consisted of one defender, two midfielders, and one striker. To maintain the intensity of the game, a spare ball and substitute players were kept at the side of the field to quickly introduce new balls when needed. In addition, each training session started with a 15 min standardized warm-up program consisting of low-intensity running and soccer-specific dynamic stretching. The description of the training protocols for the ISSG and CSSG groups are presented in Table 2.

### 2.2. Participants

Due to the small sample size, this research was considered a pilot study to gather preliminary data for future large-scale investigations. A total of 16 young male soccer players (ISSG [*n* = 8, M_age_ = 16.50 ± 0.53 years, M_height_ = 1.72 ± 0.03 m, M_body mass_ = 63.95 ± 13.26 kg, M_body mass index_ = 21.48 ± 3.81 kg/m^2^] and CSSG [*n* = 8, M_age_ = 16.63 ± 0.52 years, M_height_ = 1.73 ± 0.04 m, M_body mass_ = 55.75 ± 5.57 kg, M_body mass index_ = 18.74 ± 1.75 kg/m^2^]) were recruited from a domestic soccer club. The sample size was calculated using G*Power (version 3.1.9.4) [32]. The effect size was 0.4, the power was 0.80, and the *p* value was 0.5 for two groups and two measurements (pre-test–post-test) and the estimated total sample size was sixteen [16].

The inclusion criteria of the players were as follows: (a) participating in all pre-test and post-test measurements; (b) participating in at least 90% of the training sessions; (c) not having any injuries, illnesses, or physical limitations that would prevent participation in the study. After detailed information was given to both the players and their parents about the study, a written informed consent form was obtained from them. The study protocol was approved by Gaziantep University Sports and Health Local Ethics Committee with the decision dated 31 January 2024 and numbered 447823 (2024/03) and the study was carried out in accordance with the Declaration of Helsinki.

Randomization procedures were conducted using a stratified randomization method, which accounted for players’ pre-test performance and playing positions. Players were stratified based on their pre-test results and playing positions to ensure balanced physical characteristics and performance levels. Within each stratum, players were then randomly assigned to either the ISSG or CSSG group, maintaining the principles of randomization while achieving comparable baseline characteristics between the groups. This stratified randomization approach aimed to minimize potential confounding factors and ensure comparable baseline characteristics between the two groups.

### 2.3. Measures

Anthropometric assessment: Body height was measured using a stadiometer (SECA, GmbH, Hamburg, Germany) with an accuracy of 0.1 cm, and body mass was measured using an electronic scale (SECA, Germany) with an accuracy of 0.1 kg/m^2^. The body mass index then was calculated as weight/height squared (kg/m^2^) [16,33].

30 m sprint test: The speed of the players was assessed using the 30 m sprint test. Participants started 30 cm behind the starting line [34], with sprint times measured using timing gates equipped with photocells (e.g., Brower Timing System, Draper, UT, USA) for accuracy. Each player completed the 30 m track three times at maximum speed, with a 3 min passive rest between repetitions. The fastest time recorded by the system was used for analysis [35].

Horizontal Jump (HJ): Players start the horizontal jump test with their toes just behind the line. Legs shoulder-width apart, knees flexed, arms in front, and with the swing of the arms they tried to jump as far as possible. The distance was measured with the help of a standard tape measure placed perpendicular to the heel of the foot at the point where the players fell after the jump [36,37]. The test was performed with 2 repetitions and 2 min passive rest was given between repetitions. The best jump was recorded in centimeters.

Illinois Agility Test (IAT): The IAT tests acceleration, deceleration, reaction time, and change in direction. The course consists of four center cones spaced 3.3 m apart, with four corner cones positioned 2.5 m from the center cones. It includes a 180° turn every 10 m, a 40 m straight segment, and a 20 m section requiring changes in direction. Players begin the test lying face down behind the start line, arms at their sides and head facing forward. Photocells (Smartspeed, Fusion Sport Pty Ltd., Brisbane, QLD, Australia) are placed at the start and finish lines to record times. The test is performed three times, with 3 min of passive rest between repetitions. The best time recorded in seconds is used for analysis. Disqualification occurs if a player fails to follow the course instructions, does not reach the finish lines, or cannot complete the course [38].

Static Balance (SB): Static balance of the players was assessed using the Flamingo Balance Test. Players were instructed to stand barefoot on a board (50 cm long, 5 cm high and 3 cm wide), balance on one leg, bend their free leg at the knee, keep their foot close to the hip and stand like a flamingo by placing their hands on the iliac bone. A stopwatch was used to record the time for each trial. Players were asked to stand in the specified position for 1 min. The time was stopped when the players lost their balance and then was restarted when the player was ready. Players performed 3 trials with eyes open on each leg and the number of falls in 1 min was recorded. The average of these 3 measurements was evaluated for analysis. Unlike other testing procedures where the best score was recorded, the Flamingo Balance Test used the average of the three trials to ensure greater reliability and to account for potential variations in balance performance. Passive rest was given for 2 min between repetitions [39,40].

Aerobic Capacity: Aerobic capacity was assessed using the Yo-Yo Intermittent Recovery Test-Level 1 (YYIRT1). The YYIRT1 consists of repeated 2 × 20 m runs at progressively increasing speeds, which are controlled by audio cues from a wireless speaker. Between each running bout, participants are given a 10 s rest period, during which they must move to a cone located 5 m away before returning to the start line. The test begins with four running bouts at speeds ranging from 10 to 13 km·h^−1^, followed by seven runs at 13.5 to 14 km·h^−1^. After this initial phase, the YYIRT1 continues with incremental speed increases of 0.5 km·h^−1^ after every eight running bouts, continuing until the participant reaches exhaustion. The final distance covered in the test was recorded in meters and used as the main outcome [31]. The maximal oxygen uptake (VO2max in mL/min/kg) was estimated using the following equation: VO2max = 36.4 + 0.0084 × final distance (m) [31].

Anaerobic Capacity: Anaerobic capacity was assessed using the Repetitive Sprint Test (RAST), in which participants completed 7 sprints of 35 m with 30 s rest intervals. The RAST was conducted on a natural grass football field to replicate the real playing conditions that football players frequently encounter during training and matches. This approach was deliberately chosen by the research team to enhance the ecological validity of the study and to ensure that the results reflect the athletes’ performance in their natural sporting environment. During rest intervals, participants walked back to the start line where they began each sprint 30 cm behind the starting line in a standing position. Photocells (Smartspeed, Fusion Sport Pty Ltd., Brisbane, QLD, Australia) were placed at the start and finish lines. The total sprint time (RSAtotal) of the athletes was recorded in s for analysis [34,41]. Anaerobic power was calculated based on the Repeated Sprint Ability Test (RAST), using the formula: Anaerobic power = (body mass × distance^2^) ÷ time^3^.

Perceived Exertion Rating Scale: Perceived exertion was assessed immediately after each training session. Players individually rated their perceived exertion (RPE) on the CR-10 Borg scale [42], to ensure accurate and unbiased responses [43]. This timing allowed for the evaluation of RPE at the peak of their physical effort, reflecting the intensity of the training session.

### 2.4. Procedure

Preintervention: Before the work started, the team staff was given detailed information about the work. After the possible benefits and risks of the study were explained to the parents of the athletes in detail, they were asked to sign informed consent forms. Then, the training program was prepared together with the team coach. In order for the soccer players to adapt to the test and training protocol, a familiarization training program was applied for 2 sessions per week for 4 weeks. After the familiarization program, pre-test assessments were conducted on 3 test days with 48 h of rest in between. On the first day of the assessment, height and body weight were measured before noon. In the afternoon session of the same day, horizontal jump and Illinois agility tests were performed, respectively. On the second test day, a 30 m sprint test was performed first, followed by a repeated sprint ability test. On the last test day, the flamingo balance test and the YYIRT1 were performed, respectively. Except for anthropometric assessments (10:00 a.m.), all assessments were performed at the same time of the day (6:00 p.m.–7:00 p.m.). A 5 min rest period was given between the tests performed on the same day. Except for the anthropometric evaluations and static balance tests performed in a gym, all other tests were performed on synthetic turf field. All tests were performed under similar climatic conditions (26 °C ± 3.4 °C). Before all training sessions and tests, players underwent a standard warm-up program consisting of approximately 8 min of low-intensity running, 40–60 m of 4 min acceleration and deceleration followed by 3 min of dynamic stretching (hip extensors, hip flexors, hamstrings, and quadriceps muscles). Every training and testing session was supervised by our research team.

Intervention: After the pre-test assessment, both groups (ISSG and CSSG) performed the separately designed training program for 4 weeks (Table 2). These training sessions were supervised by our research team and the team’s coaches.

Post intervention: After the 4-week training intervention, the post-test assessments were performed in a similar space, climatic condition, and time period as in the preintervention session. After the study was completed, soccer players in both groups were given the opportunity to do the training program of the other group.

### 2.5. Statistical Analysis

Mean, and standard deviation (SD) were used to describe the data. Then, the normality and homogeneity of the data were assessed using Shapiro–Wilk and Levene tests, respectively. Skewness and kurtosis values were checked for data sets that did not show a normal distribution, and data sets within ±2 were considered to have a normal distribution [44]. A repeated measures ANOVA (2 × 2) test was used to evaluate the 30 m sprint, horizontal jump, IAT, Yo-Yo test, flamingo balance test, and anaerobic and aerobic capacity measures in both groups. In addition, an independent samples t-test was used to compare between groups differences in RPE. To maintain consistency, we used the criteria outlined by Lakens (2013) for interpreting eta-squared values. The effect size was presented as ηp^2^ for a two-way ANOVA test and interpreted using the follow criteria: minimum effect (ηp^2^ ≤ 0.02), moderate effect (0.02 < ηp^2^ ≤ 0.09), and strong effect (ηp^2^ > 0.09) [45]. Data were analyzed using Statistica software (version 13.1; Statsoft, Inc., Tulsa, OK, USA), and the significance level was set at *p* < 0.05.

## 3. Results

The 30 m sprint test: The mean for the 30 m sprint test showed no significant differences between tests (F = 2097, *p* = 0.191, ηp^2^ = 0.231), (Table 3). The repeated measures ANOVA results related to the 30 m sprint test showed there was no significant differences for a Groups × tests interactions (F = 0.052, *p* = 0.827, ηp^2^ = 0.007) and between groups (F = 0.095, *p* = 0.767, ηp^2^ = 0.013). 

**Figure 1:**
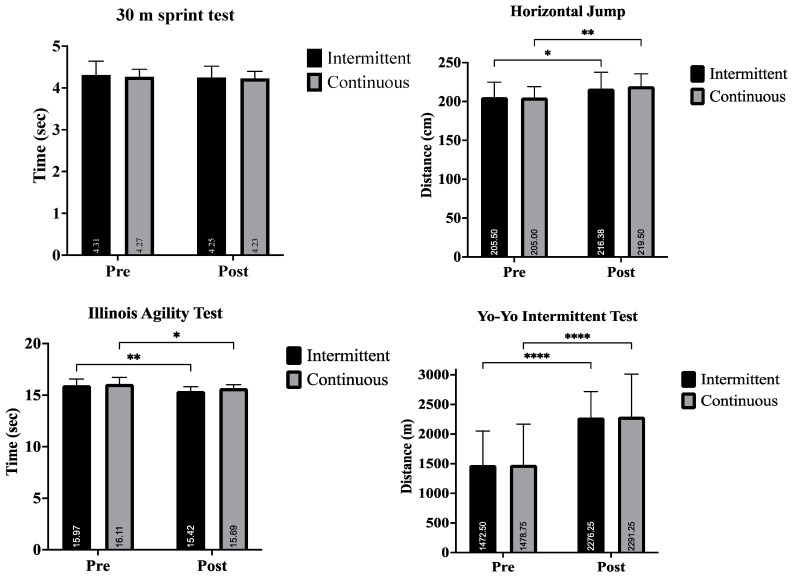

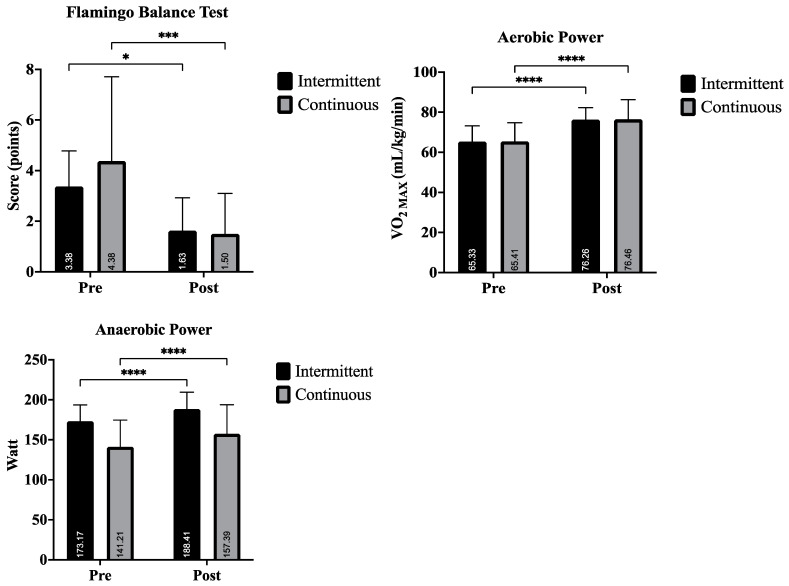
Improvement in performance responses between the groups (**** *p* < 0.0001, *** *p* < 0.001, ** *p* < 0.01, * *p* < 0.05).

Horizontal jump test: The mean for the horizontal jump test in the post-tests was significantly higher than in the pre-tests (F = 18.455, *p* = 0.004, ηp^2^ = 0.725), (Table 3). The repeated measures ANOVA results related to horizontal jump test showed there is no significant differences for a Groups × tests interaction (F = 0.431, *p =* 0.533, ηp^2^ = 0.058) and between groups (F = 0.032, *p* = 0.862, ηp^2^ = 0.005). The results are also presented in Figure 1.

Illinois agility test: The mean for the Illinois agility test in the post-tests was significantly lower than in the pre-tests (F = 70.024, *p* = 0.000, ηp^2^ = 0.909), (Table 3). The repeated measures ANOVA results related to the Illinois agility test showed there is no significant differences for a Groups × tests interaction (F = 0.225, *p* = 0.650, ηp^2^ = 0.031) and between groups (F = 0.511, *p* = 0.498, ηp^2^ = 0.068). The results are also presented in Figure 1.

Aerobic capacity: The mean for the Yo-Yo test in the post-tests was significantly higher than in the pre-tests (F = 45.870, *p* = 0.000, ηp^2^ = 0.868), (Table 3). The repeated measures ANOVA results related to the Yo-Yo test showed there is no significant differences for a Groups × tests interaction (F = 0.003, *p* = 0.957, ηp^2^ = 0. 000) and between groups (F = 0.003, *p* = 0.961, ηp^2^ = 0.000). The results are also presented in Figure 1. 

Aerobic power: The mean for the aerobic power in the post-tests was significantly higher than in the pre-tests (F = 45.848, *p* = 0.000, ηp^2^ = 0.868), (Table 3). The repeated measures ANOVA results related to aerobic power showed there is no significant differences for a Groups × tests interaction (F = 0.003, *p* = 0.957, ηp^2^ = 0.000) and between groups (F = 0.003, *p* = 0.961, ηp^2^ = 0.000). The results are also presented in Figure 1.

Anaerobic capacity: The mean for the anaerobic power in the post-tests was significantly higher than in the pretests (F = 87.786, *p* = 0.000, ηp^2^ = 0.926), (Table 3). The repeated measures ANOVA results related to anaerobic power showed there is no significant differences for a Groups × tests interaction (F = 0.104, *p* = 0.757, ηp^2^ = 0.015) and between groups (F = 3.167, *p* = 0.118, ηp^2^ = 0.311). The results are also presented in Figure 1.

Flamingo balance test: The mean for the flamingo balance test in the post-tests was significantly lower than in the pre-tests (F = 42.781, *p* = 0.001, ηp^2^ = 0.811), (Table 3). The repeated measures ANOVA results related to the flamingo balance test showed there is no significant differences for a Groups × tests interaction (F = 1.340, *p* = 0.285, ηp^2^ = 0.161) and between groups (F = 0.462, *p* = 0.519, ηp^2^ = 0.062). The results are also presented in Figure 1.

RPE values were assessed immediately after each training session and are presented in Table 4, which compares the mean values between the ISSG and CSSG groups.

**Figure 2:**
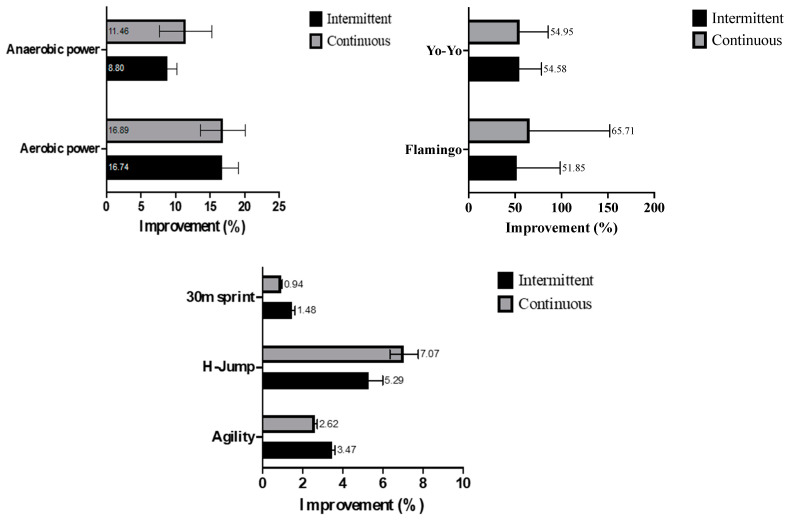
Improvement in aerobic and anaerobic performance, physical and technical performance responses between the trials.

In addition, Figure 1 and Figure 2 illustrate the improvements and percentage gains in aerobic and anaerobic performance, as well as physical and technical performance responses observed between the ISSG and CSSG trials. Technical performance responses refer to improvements in decision-making, spatial awareness, and teamwork skills observed during training sessions. The data highlights significant advancements in areas such as aerobic and anaerobic capacity, flamingo, Yo-Yo intermittent test, Illinois agility test, horizontal jump, and 30 m sprint performance.

## 4. Discussion

The aim of this study was to compare the effects of intermittent ISSG and CSSG on athletic performance metrics, including aerobic and anaerobic capacity, agility, speed, horizontal jump, and static balance, in young soccer players. This study is considered a pilot study due to the small sample size. Therefore, the results should be interpreted with caution, and future research with larger sample sizes is needed to confirm these preliminary findings. The results showed that both ISSG and CSSG significantly improved these parameters, with no significant differences observed between the two groups, except for higher RPE values in the CSSG group. These findings suggest that both training modalities are effective in enhancing physical performance attributes in youth soccer players.

In our study, a significant difference was found in both groups in the post-test compared to the pre-test in terms of VO2max and Yo-Yo test results. However, it was found that there was no significant difference between the groups. Similarly, the effects of CSSG consisting of a single set of 25–40 min and ISSG applied for 5 min, 5–8 sets with 1 min rest intervals were compared [16]. Their analysis found no significant difference between the groups in terms of VO2max and Yo-Yo test results. However, when the pre-test and post-test results were compared, it was determined that there was a significant increase only in the ISSG group. In another study, ISSG and CSSG formats involving 2 vs. 2, 3 vs. 3, and 4 vs. 4 games were compared, and similar physiological responses were found in both groups [46]. One study also showed that small-sided games (SSG) increased aerobic power [47]. In contrast, our findings align with those of [48,49], who reported that SSGs are effective in improving aerobic performance in young football players. These results highlight the versatility of SSGs in enhancing various aspects of physical performance, particularly aerobic capacity, which is crucial for overall athletic development. This evidence further supports the implementation of SSGs as a practical training method for optimizing both physical and tactical performance. In the following section, we explore how these findings translate into game-specific contexts and their implications for long-term player development. A study found that both CSSG and ISSG caused a decrease in the body mass of football players, but this decrease was not significant. By comparing these improvements, it is evident that both ISSG and CSSG can enhance physical performance attributes. Coaches can use this insight to tailor training programs to optimize player development based on specific objectives. The researchers attribute the lack of significant reduction to the initial body weight and the physical fitness levels of the players [16].

In our study, repeated sprint ability, which reflects anaerobic capacity, results improved significantly in the post-test compared to the pre-test. Arslan et al. [48], reported that repeated sprint ability performance increased significantly by 9.5% at the end of the study. Eniseler et al. [50] applied SSG and RST exercises to two different groups for approximately 60 to 90 min and found that the repeated sprint ability of both groups improved. In addition, it was also reported that the development effect of SSG was greater. In addition, there was no significant difference in speed values in both groups in the post-test compared to the pre-test. Similarly, no significant difference was found between the groups. One study also showed that speed increased in post-test measurements compared to pre-test in both groups and this increase was greater in the ISSG training group, but this increase was not significant [16]. In parallel with this study, the lack of significant improvement in certain fitness attributes, such as 30 m sprint speed, may be attributed to the initial training level and specificity of the intervention protocol. The participants were already trained soccer players with a baseline level of sprinting ability, which may have limited the potential for further improvements over the relatively short duration of the study (four weeks). Additionally, the nature of the training sessions, which focused on SSG, may not have provided the specific, high-intensity stimulus necessary to elicit significant gains in maximal sprint performance. SSGs are known to improve aerobic capacity and agility, but their direct impact on sprint performance can be limited due to the intermittent nature of the activity and the inclusion of technical and tactical demands. Furthermore, the relatively low frequency of training (two sessions per week) may not have been sufficient to induce significant neuromuscular adaptations required for sprint performance improvements. Future studies could consider integrating additional sprint specific drills or increasing training frequency to better address this fitness attribute.

Regarding the agility performance, an increase was observed in the post-test measurements compared to the pre-test in our study. However, this increase did not show a significant difference between the groups. On the contrary, in the study that was conducted, no significant difference was found between pre-test and post-test agility values [16]. The reason for the lack of significant difference in this study compared to our study may be the age of the subjects, initial physical fitness level, and training experience. As a matter of fact, it is thought that SSG training is effective for improving agility, but there may possibly be an age or technique limitation [51]. Similar to our findings, there are also studies showing that ISSG training improves agility [48,52]. A meta-analysis, suggests that SSGs performed 2–3 days a week can enhance agility in team sports [53]. Based on our findings, both ISSG and CSSG can contribute to agility development, though further research is needed to determine which type is more effective.

The post-test horizontal jump (HJ) measurements of both ISSG and CSSG groups were significantly higher than their pre-test measurements, indicating improvements in lower limb explosive strength. Similar studies, such as [48,54], have reported that small-sided game (SSG) interventions enhance performance in countermovement jump (CMJ) and squat jump (SJ) tests. These findings suggest that SSGs effectively develop neuromuscular adaptations by engaging players in repeated high-intensity actions and multi-directional movements. However, the differences between HJ, CMJ, and SJ may arise from their distinct biomechanical demands; HJ emphasizes horizontal propulsion, often reflecting sprint-related capabilities, while CMJ and SJ focus on vertical force generation, which is crucial for vertical jump performance. This highlights the importance of test selection in evaluating specific physical attributes, as different jump types provide unique insights into an athlete’s explosive strength and power profile [55].

In the present study, the RPE values were significantly higher in the CSSG group compared to the ISSG group (*p* < 0.05). This aligns with findings from [16], where continuous small-sided games led to increased fatigue levels due to the absence of rest intervals. These results suggest that CSSG imposes greater cardiovascular and muscular demands, which may be beneficial for aerobic endurance development but requires careful monitoring to avoid overtraining. In another study, RPE values were similar in both groups [56]. In addition, one of the most important variables to be considered in relation to RPE is the field size. Most studies suggest that RPE increases with increasing field size [57,58,59]. In this line, a significant improvement was obtained in the post-test measurements compared to the pre-test in both groups. However, no significant difference was observed between the groups. Similar to the results of our study, although many studies have reported that SSGs positively affect balance development [49,52,60], no study has examined the effects of different SSG types (ISSG and CSSG) on balance development. Therefore, further studies examining balance performance with different types of SSG may reveal the potential advantages and disadvantages of these training methods.

This study is a comprehensive investigation into the effects of ISSG and CSSG methods on the physical performance of young soccer players. However, the following points should be highlighted to provide a broader perspective on the findings: Firstly, the sample size was determined using G*Power analysis, demonstrating that the sample size was sufficient to ensure the validity of the study findings. In this study, dietary habits were assessed based on participants’ self-reported information, which was combined with regular exercise monitoring. Tracking dietary and exercise routines minimized individual variability, leading to more reliable results. Nonetheless, future studies could benefit from more detailed dietary monitoring, such as daily food intake records or biochemical analyses, to further reduce variability. Although the intervention period was limited to 4 weeks, this duration was sufficient to evaluate the short-term effects of ISSG and CSSG. However, longer-term studies are recommended to better understand the sustained effects of these training protocols. Extended intervention durations could provide more comprehensive insights into neuromuscular adaptations and the lasting impact of these methods on performance. Lastly, training intensity and player workload were regularly monitored using the CR-10 Borg Rating of Perceived Exertion scale in this study. Future research could incorporate tools such as GPS technology and heart rate monitors to enable a more detailed analysis of training load and physiological responses. Additionally, exploring the effects of ISSG and CSSG on different age groups, genders, and skill levels could expand the generalizability of these methods across diverse playing contexts. This study demonstrates that ISSG and CSSG are effective strategies for improving the physical performance of young soccer players. Coaches can tailor these protocols to meet the needs and training objectives of their players. Specifically, combining intermittent and continuous formats can optimize recovery, support physical development, and enhance player engagement in training programs.

## 5. Conclusions

As this research represents a pilot study, our findings indicate that both the ISSG and CSSG approaches led to improvements in athletic performance metrics in youth male soccer players. However, these preliminary results require validation through studies with larger samples. However, the lack of significant differences between the two methods suggests that coaches can select either approach based on their specific training objectives and available resources. These results emphasize the importance of evidence-based practices and highlight the need for coaches to tailor training programs to the individual needs, fitness levels, and developmental stages of their players. To optimize athletic development and performance outcomes, coaches should consider integrating both ISSG and CSSG into their training programs, while continuously monitoring player responses to ensure effective progression.

## Figures and Tables

**Table 1:** Study timeline.

	2024											
	Pre-Season				In-Season							
Months	March				April				May			
Week	1	2	3	4	5	6	7	8	9	10	11	12
Intermittent	Pre				ISSGs				Post			
Continuous	Pre				CSSGs				Post			

**Table 2:** Description of the 4-week training programs.

Weeks	Sessions	Intermittent	Continuous
1	Pre-Tests		
	1	-4 vs. 4 small-sided games	-4 vs. 4 small-sided games
2	2	-4 × 4 min bouts	-1 × 16 min bouts
		-2 min passive rest between bouts	-No rest
	3	-4 vs. 4 small-sided games	-4 vs. 4 small-sided games
3	4	-4 × 5 min bouts	-1 × 20 min bouts
		-2 min passive rest between bouts	-No rest
	5	-4 vs. 4 small-sided games	-4 vs. 4 small-sided games
4	6	-4 × 6 min bouts	-1 × 24 min bouts
		-2 min passive rest between bouts	-No rest
	7	-4 vs. 4 small-sided games	-4 vs. 4 small-sided games
5	8	-4 × 7 min bouts	-1 × 28 min bouts
		-2 min passive rest between bouts	-No rest
6	Post-Tests		

Note: During passive rest periods, players remained on the field in a seated or standing position to allow recovery without engaging in active movements. They were encouraged to hydrate and could receive brief tactical feedback from coaches. This ensured adequate recovery while maintaining readiness for the next bout.

**Table 3:** Pre- and Post-Test performance differences in both groups.

Variables			Tests		Groups × Tests	
	Pre (M ± SD)	Post (M ± SD)	*p*	η_p_^2^	*p*	η_p_^2^
30 m (s)	(G1) 4.31 ± 0.33	4.25 ± 0.27	0.191	0.231	0.827	0.007
	(G2) 4.27 ± 0.18	4.23 ± 0.17				
HJ (cm)	(G1) 205.50 ± 19.32	216.37 ± 21.17	0.004 *	0.725	0.533	0.058
	(G2) 205.00 ± 14.06	219.50 ± 16.04				
IAT (s)	(G1) 15.97 ± 0.60	15.42 ± 0.41	0.000 **	0.909	0.650	0.031
	(G2) 16.11 ± 0.63	15.68 ± 0.34				
Yo-Yo (m)	(G1) 1472.50 ± 577.55	2276.25 ± 439.02	0.000 **	0.868	0.957	0.000
	(G2) 1478.75 ± 687.45	2291.25 ± 720.15				
Flamingo (score)	(G1) 3.37 ± 1.41	1.62 ± 1.30	0.001 **	0.811	0.285	0.161
	(G2) 4.37 ± 3.33	1.50 ± 1.60				
Anaerobic power	(G1) 173.17 ± 20.44	188.41 ± 21.06	0.000**	0.926	0.757	0.015
	(G2) 144.21 ± 33.45	157.38 ± 36.45				
Aerobic power	(G1) 65.33 ± 7.85	76.26 ± 5.97	0.000 **	0.868	0.957	0.000
	(G2) 65.41 ± 9.35	76.46 ± 9.80				

G1: Group 1, G2: Group 2. 30 m—30 m sprint test, HJ—horizontal jump test, IAT—Illinois agility test, Flamingo—Flamingo balance test, Anaerobic Power: Anaerobic power (body mass × distance^2^ ÷ time^3^), Aerobic Power: Aerobic power (mL·min^−1^·kg^−1^). Distance total distance obtained from RAST * Denotes significance at *p* < 0.05. ** Denotes significance at *p* < 0.01. Note: Groups × tests refers to the interaction between group assignments (ISSG, CSSG) and testing times (pre-test and post-test).

**Table 4:** Mean and standard deviation of changes at RPE in both groups.

Groups	Mean	Std. Dev	t	*p*
Intermittent	4.97	0.55	−1.884	0.080
Continuous	5.48	0.54		

## Data Availability

The original contributions presented in this study are included in the article. The raw data supporting the conclusions of this article will be made available by the authors on request.
